# Risk of Severe Infection among Rheumatoid Arthritis Patients on Biological DMARDs: A Population-Based Cohort Study

**DOI:** 10.3390/jcm11112955

**Published:** 2022-05-24

**Authors:** Mattia Bellan, Lorenza Scotti, Daniela Ferrante, Elisa Calzaducca, Giulia Francesca Manfredi, Pier Paolo Sainaghi, Francesco Barone-Adesi

**Affiliations:** 1 Department of Translational Medicine (DiMet), Università del Piemonte Orientale (UPO), Via Solaroli 17, 28100 Novara, Italy; mattia.bellan@med.uniupo.it (M.B.); lorenza.scotti@med.uniupo.it (L.S.); daniela.ferrante@med.uniupo.it (D.F.); 10034336@studenti.uniupo.it (E.C.); 20030060@studenti.uniupo.it (G.F.M.); francesco.baroneadesi@uniupo.it (F.B.-A.); 2 Department of Internal Medicine and Rheumatology Unit, Azienda Ospedaliero-Universitaria Maggiore Della Carità, 28100 Novara, Italy; 3 Center for Translational Research on Autoimmune and Allergic Diseases (CAAD), Università del Piemonte Orientale (UPO), 28100 Novara, Italy

**Keywords:** rheumatoid arthritis, biological DMARDs, severe infection

## Abstract

Biological disease-modifying anti-rheumatic drugs (bDMARDs) are widely used for the management of rheumatoid arthritis, although their benefits are counterweight by an increased risk of infections. In the present study, we used administrative data to compare the risk of severe infections among different classes of bDMARDs. A retrospective cohort study was conducted using Administrative Health Databases of the Piedmont Region, Italy. Relevant data were obtained from: (1) the inhabitants registry, (2) hospital discharge records, and (3) the co-payment exemption registry and (4) drug claims registry. Fine and Gray competing risk models were fitted to evaluate the association between the use of different types of bDMARDs and occurrence of severe infection accounting for treatment interruption as competing risk. A total of 1780 new users of bDMARDs were identified. Among them, 50 hospitalizations for infection occurred during the study period. The use of Tocilizumab was associated with an increased risk of infection, compared to tumor necrosis factor (TNF) inhibitor drugs (sub-distribution hazard ratios-sHR: 2.510; 95% CI: 1.279–4.926), whereas no difference in the risk of severe infection was found for abatacept (sHR: 0.584; 95% CI: 0.234–1.457). bDMARDs treatment is generally safe in clinical practice with slight but important differences among classes. The increased risk of infection associated with tocilizumab use should be taken into account when balancing the risk and benefits of starting a treatment with this drug.

## 1. Introduction

In 1999, a trial firstly proved the clinical benefit of adding etanercept, a tumor necrosis factor inhibitor (TNFi), to methotrexate in Rheumatoid Arthritis (RA) patients who did not respond to the standard of care [1]. Since that cutting-edge moment, 10 biological diseases modifying antirheumatic drugs (bDMARDs) have been approved for the treatment of RA, and many others are under investigation [2]. The current recommendations for the management of RA, lastly released in 2019, advise the use of bDMARDs in patients who have not achieved the target of treatment with classical synthetic DMARDs (csDMARDs) [3]. RA is the most common chronic inflammatory disease, with an estimated prevalence worldwide ranging from 0.24 to 0.72% [4,5]. As the rate of non-responders to csDMARDs is estimated to be around 40% [6], bDMARDs are being prescribed to a larger and larger number of patients. This recently led to some controversy in the use of these medications: indeed, different cost-effectiveness studies lead to conflicting results, given that it is hard to reliably counterbalance the direct costs of the drugs with the indirect beneficial effects, particularly with the limitation of RA-related morbidity and disability [7]. Beside the economic burden, bDMARDs prescription is also associated with clinical risks, in particular a higher incidence of infections. However, it is still not clear whether this risk differs among different bDMARDs [8]. In this study, we evaluated the safety of bDMARDs in RA patients with respect to the risk of severe infection using the administrative data of a large Italian region, an approach which has already been demonstrated to be useful in this context [9].

## 2. Materials and Methods

A retrospective cohort study was conducted using the administrative health databases of Piedmont Region, Italy (about 4,400,000 inhabitants, corresponding to 7.5% of the national population). Relevant data were obtained from: (1) the inhabitants registry (containing demographic information such as gender, date of birth, date of death, etc.); (2) hospital information system, including hospital discharge records from public or private hospitals; (3) co-payment exemption registry; (4) and drug claims registries, including records of the drug prescriptions reimbursable by the Italian National Health Service dispensed by hospital and local pharmacies. These databases can be linked through a unique encrypted identifier and have largely been used for epidemiological research [10,11,12]. All procedures conducted in this study were in accordance with the ethical standards of the institutional and/or national research committee and with the 1964 Helsinki Declaration and its later amendments. The study protocol was approved by the local ethical committee of the “Maggiore della Carità” Hospital, Novara. The study was conducted using data routinely collected in the aforementioned regional administrative health care databases in which authors had access to anonymized data only, hence, informed consent was not required. The target population consisted of all subjects residing in Piedmont affected by RA who received at least a prescription of bDMARD (Anatomical Therapeutic Chemical (ATC) codes: L04AB01, L04AB04, L04AB02, L04AB06, L04AB05, L04AC07, L04AA24) between 1 January 2013 to 31 December 2017. The first prescription during this period was defined as the index prescription and the corresponding dispensation date as index date. Subjects were defined as affected by RA according to the algorithm developed by Carrara et al. [13], which takes into account hospitalization with discharge diagnosis of RA, co-payment exemption for RA, and prescriptions of anti-rheumatic drugs. To ensure the inclusion of only incident users, we excluded subjects who received prescriptions of bDMARDs in the year preceding the index date (1-year look-back). Subjects were followed from the index date until the earliest date among hospitalization for severe infection, death or end of follow-up (31 December 2018). According to previous studies, the hospital discharge database was used to identify the cohort members who were hospitalized with primary or secondary diagnosis of severe infections, namely meningitis/encephalitis (ICD-9 codes: 003.21, 036.0, 047.*, 049.*, 053.0, 054.72, 072.1, 091.81, 094.2, 098.82, 100.81, 320.*, 036.1, 054.3, 056.01, 058.21, 058.29, 062.*, 063.*, 064.*, 066.41, 072.2, 094.81, 130.0, 323.*), cellulitis / soft tissue infection (ICD-9 codes: 035, 040.0, 569.61, 681.*, 682.*, 728.86, 785.4), pneumonia (ICD-9 codes: 003.22, 480.*, 481, 482.*, 483.*, 484.*, 485.*, 486.*, 487.0), pyelonephritis (590.*), septic arthritis/osteomyelitis (ICD-9 codes: 003.23, 056.71, 098.5*, 711.0, 711.00–711.07, 711.09, 711.9*, 003.24, 376.03, 526.4, 730.0*, 730.1*, 730.2*), endocarditis (ICD-9 codes: 036.42, 074.22, 093.2*, 098.84, 421.*, 422.92), and bacteraemia/sepsis (ICD-9 codes: 038.*, 790.7, 995.91, 995.92) [14]. All bDMARDs prescriptions received during follow-up by cohort members were considered. Patients were classified as users of inhibitors of tumour necrosis factor drugs (TNFi ATC codes: etanercept L04AB01, infliximab L04AB02, adalimumab L04AB04, certolizumab L04AB05, golimumab L04AB06), tocilizumab (the only anti-interleukin 6 available, ATC code: L04AC07) and abatacept (ATC code: L04AA24). Treatment discontinuation was also considered and defined as a gap of at least 90 days between the end of the coverage of a prescription and the beginning of the subsequent one. The discontinuation date was defined as the date of the end of coverage of the last prescription. The use of csDMARDs (ATC codes: methotrexate L01BA01, L04AX03, sulfasalazine A07EC01, leflunomide L04AA13), choloroquine or hydroxichloroquine (ATC codes: P01BA01, P01BA02) was assessed in the year before cohort entry to take into account RA treatment received before start of bDMARDs therapy. Moreover, the following potential confounders were measured at cohort entry: age, sex, and Charlson comorbidity index [15]. Finally, the use of corticosteroids (ATC code:H02*) and hospitalization for interstitial lung disease (ICD-9 codes: 516.3, 517.2, 517.8, 714.81, 515.*, 135.*,495.*) [16] was assessed during the entire follow-up of the study. Descriptive statistics were used to summarize the main characteristics of cohort members. Categorical variables were reported as absolute frequencies and percentages, and continuous variable as mean and standard deviation (SD). Chi square or exact tests were used to assess the association between patients’ characteristics and type of bDMARDs used at cohort entry. Univariable and multivariable Fine and Gray competing risk models were fitted to estimate the sub-distribution hazard ratios (sHR) and their corresponding 95% confidence intervals (95% CI) for the association between type of bDMARDs and risk of serious infections, accounting for treatment discontinuation as competing event. Cumulative incidence curves derived from the univariable Fine and Gray model were drawn to describe the cumulative incidence of severe infection according to first bDMARD used. Multivariable model included age, sex, Charlson comorbidity index, onset of interstitial lung disease, and use of corticosteroids as potential confounding factors. Type of bDMARD, onset of interstitial lung disease, and use of corticosteroids during follow-up were included in the regression model as time-dependent variables. All tests were two tailed and type I error was set to 0.05. Statistical analyses were performed using SAS version 9.4 (SAS Institute Inc., Cary, NC, USA).

## 3. Results

**Figure 1:**
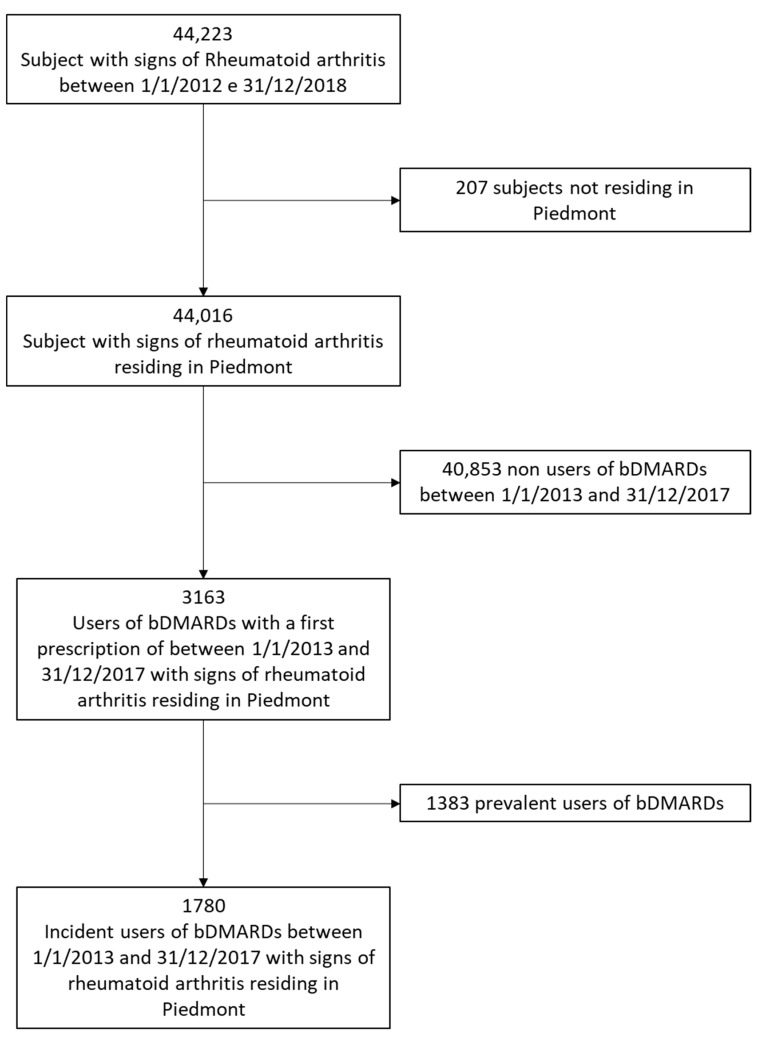
Flow chart. bDMARD: biological disease-modifying anti-rheumatic drugs.

Figure 1 shows the flow-diagram of the cohort exclusion criteria. According to the algorithm of Carrara et al. [13], 44,223 subjects were affected by RA in Piedmont between 1 January 2012 and 31 December 2018. From this initial cohort, 207 subjects were excluded due to not residing in the region and 40,853 because they did not receive any bDMARDs prescription between 1 January 2013 and 31 December 2017. Finally, 1383 subjects were excluded due to being prevalent users of bDMARDs. The final cohort thus consisted of 1780 subjects.

Table 1 reports the distribution of subjects’ characteristics according to the type of bDMARD received at cohort entry. Subjects treated with abatacept were older and more frequently women compared to the other treatments. Almost all patients treated with abatacept and TNFi at cohort entry had been treated with csDMARDs or chloroquine in the year before cohort entry, whereas a smaller proportion was observed for users of tocilizumab (65.49%). Moreover, abatacept users were more frequently treated with corticosteroids (82.71%) compared to the other treatments. Finally, subjects treated with tocilizumab were in worse clinical conditions at the beginning of the treatment than the others (proportion of subjects with Charlson comorbidity index ≥ 1:20.42%). Cohort members accumulated 3574.67 years of follow-up and generated 49 events of serious infections with an incidence rate of 1.37 per 100 person-years.

**Figure 2:**
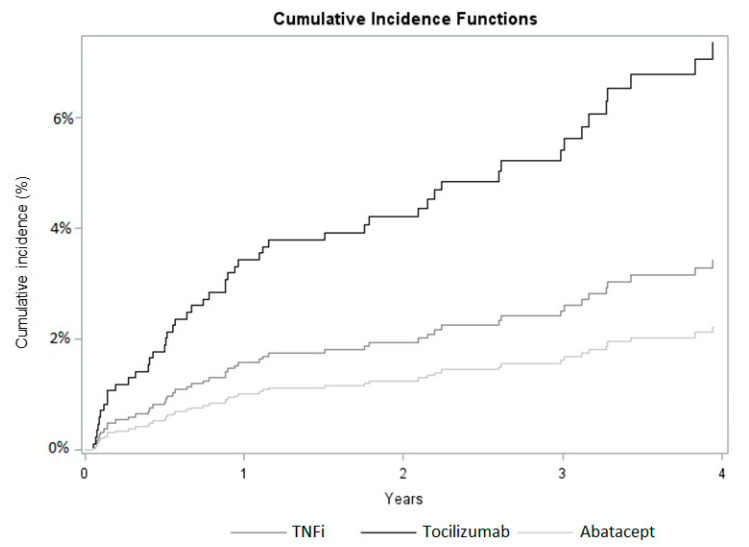
Cumulative incidence functions of severe infections stratified by bDMARD used at cohort entry. Estimates derived by the Fine and Gray model.

Figure 2 shows the cumulative incidence function for severe infections derived from the univariable Fine and Gray model according to the type of bDMARD used at cohort entry, and Table 2 shows the cumulative incidence during the follow-up. The curves show that, among those who did not discontinue bDMARDs treatment, the baseline users of tocilizumab had the greatest cumulative incidence of severe infection, followed by TNFi and abatacept users, with cumulative incidence at 4 years equal to 7.37%, 3.44% and 2.22%, respectively.

Table 2 shows the sub-distribution hazard ratio and the corresponding 95% confidence intervals for the association between the type of bDMARDs and the risk of severe infection derived from the univariable and multivariable models. The results of the univariable model showed that among subjects who did not discontinue bDMARD therapy, those treated with tocilizumab had a risk of developing a severe infection that was 2.336 times higher than those treated with TNFi, whereas no difference in the risk of severe infection was found between abatacept and TNFi users. The sHR remained substantially unchanged after the adjustment for the selected potential confounders, confirming that tocilizumab users had a statistically significant increased risk of developing severe infections compared to TNFi users (sHR 2.493, 95% CI: 1.275–4.876).

## 4. Discussion

The use of bDMARDs in clinical practice is nowadays largely widespread. One of the most relevant concerns related to their use is the risk of severe infections, which may vary among the different biological target addressed. In this paper, using the administrative data of a large Italian region, we found that tocilizumab was associated with a higher risk of infections than abatacept and TNFi. In 2010, the European League Against Rheumatism (EULAR) released the first version of the recommendations for the management of RA with synthetic and biological DMARDs [17]. This document was further updated in 2013, 2016, and 2019. Every version was informed by ad hoc systematic reviews that confirmed a good safety profile for bDMARDs [18,19,20,21]. In the last report, the authors reported that the risk of severe infections is moderately increased in users of bDMARDs compared to those receiving csDMARDs. This result is in line with a recent Danish cohort study that demonstrated a hazard ratio for serious infections of 3.7 (95% CI 3.4–4.1) for bDMARDs users compared to non-users [22]. Studies also suggest that this risk would be higher during the first year after treatment initiation and that patients with a first episode of infection are at a higher risk for subsequent events [22,23,24]. Although the increased risk of severe infection in bDMARDs users seems to be established, whether this risk differs among the different drugs of this class is more controversial. According to Sepriano et al., who systematically reviewed 13 papers comparing the risk for severe infections among different bDMARDs, there is no major difference among groups [21]. Consistent with this meta-analysis, Jeon et al. recently published a comparison between tocilizumab users and TNFi users in a large retrospective Korean cohort study [25]. According to the authors, the two groups did not differ in terms of global risk of severe infections, but tocilizumab was associated with a higher risk of skin and subcutaneous tissue infections and a lower risk of urological and gynecological infections compared to TNFi. Similarly, in a previous report, the use of tocilizumab was not associated with a higher risk of composite severe infections than TNFi [26]. However, the use of tocilizumab was associated with an increased risk of specific infections, such as serious bacterial infection, skin and soft tissue infections, and diverticulitis if compared to TNFi. When compared to abacept, tocilizumab use was associated with an increased risk of composite severe infections, serious bacterial infection, diverticulitis, pneumonia/upper respiratory tract infection, and septicaemia/bacteraemia [24]. In a work based on a Japanese registry of RA patients treated with biologic drugs, Sakai et al. directly compared the rate of severe infections between the administration of TNFi and tocilizumab [27]. A first comparison showed a significantly higher infection rate in the tocilizumab group than in the TNFi group. However, the two groups were not perfectly comparable with respect to concomitant treatment, age, RA disease stage, and comorbidities prevalence. After the adjustment for these covariates, no difference concerning the infection risk between the two groups emerged [27]. On the other side, some reports suggest that abatacept might be associated with a lower risk of infections. Carrara et al. published a study based on administrative data retrieved from 4,656 RA patients with at least one bDMARD prescription in Lombardy Region, Italy [28]. That study was comparable to ours with regards to the geographic setting and the adopted methods and reported that abatacept was associated with a lower risk of infection than etanercept [28]. Furthermore, a large observational cohort study in the USA reported a significantly lower risk of infection for abatacept compared to other bDMARDs [29]. Although we found a reduced risk of infection among abatacept users, the results did not reach the statistical significance due to the small number of events in this group. Thus, we were not able to confirm this observation. A better understanding of the relative risk of infection might contribute to a tailored approach in RA management. In fact, we still lack predictive models assisting the choice of a specific bDMARD. When deciding which drug should be prescribed, clinicians generally take into consideration several aspects, including the features of the disease along with characteristics of the patient (e.g., age, comorbidity, concomitant therapy, disease activity). Indeed, in addition to the type of bDMARD used, the dosage regimen may also significantly impact on the risk of infection. For example, the meta-analysis of Campbell et al. analyzed six trials on the incidence of adverse effects, such as infection and severe infection, in patients treated with low (4 mg/kg) or high dosage (8 mg/kg) of tocilizumab in association with methotrexate [30]. The main finding was a small but significantly increased risk of adverse events, including infection, in patients treated with high dose of tocilizumab plus methotrexate compared to the low-dose group. The increased risk of infection in our population of TCZ users may be related to the TCZ mechanism of action that inhibits the Il-6 receptor. Il-6 is relevant in the immune response to pathogens, in particular to bacteria; on the other hand, it should be noted that TCZ, in clinical practice, has mainly been used as a second biologic agent, in particular after the failure of a TNFi, thus, in patients with an increased risk of immunosuppression. We acknowledge that our study was not able to evaluate the safety of rituximab and JAK inhibitors since too few prescriptions of these drugs were available during the study period. In the case of JAK inhibitors, this is also due to the fact that their use in the clinical practice is relatively recent. The results of the multivariable model show that age, Charlson comorbidity index, and concomitant use of corticosteroids were associated with the risk of severe infections. Age is a well-known risk factor for infections [22,31,32], thus, raising some concerns about the use of bDMARDs in the elderly. However, the overall effectiveness in patients affected by young-onset and elderly-onset RA (EORA) is similar [33]; moreover, recent data demonstrate the safety of the treat-to-target strategy also in EORA patients, supporting the use of bDMARDs in the older age groups [34]. Indeed, the use of bDMARDs generally leads to a reduction in steroid dosage, which in turn is particularly relevant in this context. In fact, the chronic use of corticosteroids is responsible for an increase in infection risk, even when used at lower than 5 mg/day [35]. The use of administrative data has well-known limitations but it also some advantages, including the possibility of analyzing large, unselected cohorts of patients treated according to the current clinical practice [36]. We acknowledge the fact that patients’ administration of bDMARDS may have been biased according to local clinical practice, however, the drug prescriptions were in line with current and past guidelines [3,17,18,19,20,21], taking in consideration that reliable biomarkers for drug allocation to patients are still lacking [37,38]. The results of these kinds of studies can thus complement those coming from registry and clinical trials. Another limitation of the present study lies in the fact that, despite the large population involved, the number of events was too small to allow infection site-specific analysis. Moreover, we were not able to evaluate the effect of different dosage regimens and to adjust for potential confounders such as disease activity score and disease duration, since this information is not available. However, since corticosteroids are usually prescribed to patients with high disease activity, the association estimates can be considered as adjusted for a proxy of disease activity. Moreover, although the date of RA diagnosis is not available, all subjects included in the cohort are new users of bDMARDs, usually prescribed as second line therapy, therefore, subjects should be homogeneous in terms of disease progression, even if the onset of the disease could have occurred at different times before cohort entry. A future multi-regional study is then warranted to thoroughly evaluate the risk profile of these patients. 

## 5. Conclusions

In conclusion, our study confirms that bDMARDs treatment is generally safe in clinical practice with slight but important differences among classes. In particular, tocilizumab is associated with a greater risk of infection, along with age, comorbidities, and use of steroids.

## Figures and Tables

**Table 1:** Distribution of subjects’ characteristics according to biological disease-modifying anti-rheumatic drugs (bDMARD) used at cohort entry.

	Abatacept	Tocilizumab	TNFi	Total	*p*-Value
	(N = 295)	(N = 142)	(N = 1343)	(N = 1780)	
**Variable**					
**Age,** mean (SD)	60.19 (13.62)	52.84 (15.84)	50.94 (17.58)	52.63 (17.19)	<0.0001
**Sex**					
*Females*	246 (83.39)	110 (77.46)	937 (69.77)	1293 (72.64)	<0.0001
*Males*	49 (16.61)	32 (22.54)	406 (30.23)	487 (27.36)	
**Drug use one year before cohort entry**					
*csDMARDs*	246 (83.39)	93 (65.49)	1229 (91.51)	1568 (88.09)	<0.0001
*Corticosteroids*	244 (82.71)	114 (80.28)	967 (72.00)	1325 (74.44)	0.0002
**Charlson comorbidity index**					
*0*	251 (85.08)	113 (79.58)	1276 (95.01)	1640 (92.13)	<0.0001
*≥1*	44 (14.92)	29 (20.42)	67 (4.99)	140 (7.78)	
**bDMARD at cohort entry**					
*Abatacept*	295 (100.00)	-	-	295 (16.57)	
*Etanercept*	-	-	572 (42.59)	572 (32.13)	
*Infliximab*	-	-	66 (4.91)	66 (3.71)	
*Adalimumab*	-	-	512 (38.12)	512 (28.76)	
*Certolizumab pegol*	-	-	90 (6.70)	90 (5.06)	
*Golimumab*	-	-	103 (7.67)	103 (5.79)	
*Tocilizumab*	-	142 (100.00)	-	142 (7.98)	
**Interstitial lung disease**					
*No*	286 (96.95)	133 (93.66)	1327 (98.91)	1746 (98.09)	<0.0001
*Yes*	9 (3.05)	9 (6.34)	13 (1.19)	34 (1.91)	
**Treatment interruptions**	146	80	724	950	
**Number of events**	5	8	36	49	
**Event type ^**					
*Pneumonia*	3 (60.00)	4 (50.00)	10 (27.78)	17 (34.69)	0.2605 *
*Meningitis/encephalitis*	0 (0.00)	1 (12.50)	2 (5.56)	3 (6.12)	
*Bacteraemia/sepsis*	0 (0.00)	0 (0.00)	12 (33.33)	12 (24.49)	
*Cellulitis/soft-tissue infections*	0 (0.00)	1 (12.50)	5 (13.89)	6 (12.24)	
*Endocarditis*	0 (0.00)	1 (12.50)	1 (2.78)	2 (4.08)	
*Pyelonephritis*	1 (20.00)	1 (12.50)	3 (8.33)	5 (10.20)	
*Septic arthritis/osteomyelitis*	1 (20.00)	0 (0.00)	3 (8.33)	4 (8.16)	
**Person years**	536.8	214.4	2823.47	3574.67	
**Incidence Rate per 100 py**	0.93	3.73	1.28	1.37	

^ percentages calculated on the number of events within each group * *p*-value of the Freedman–Halton exact test, TNFi: tumor necrosis factor inhibitors, csDMARDS: conventional synthetic disease-modifying anti-rheumatic drugs, SD: standard deviation, py: person-year.

**Table 2:** Sub-distribution hazard ratios and corresponding 95% confidence intervals for the association between biological disease-modifying anti-rheumatic drugs (bDMARD) and risk of severe infections.

	Univariable Model	Mutivariable Model
	sHR (95% CI)	sHR (95% CI)
**bDMARDs**		
*TNFi*	1	1
*Abatacept*	0.731 (0.307–1.743)	0.610 (0.247–1.508)
*Tocilizumab*	2.336 (1.207–4.521)	2.493 (1.275–4.876)
**Age**		1.023 (1.000–1.047)
**Sex (Females vs. Males)**		0.796 (0.413–1.534)
**Corticosteroids**		3.490 (1.786–6.818)
**Interstitial lung disease**		3.957 (1.196–13.096)
**Charlson comorbidity index**		1.718 (1.334–2.211)

TNFi: tumor necrosis factor inhibitors, sHR: sub-distribution hazard ratio, 95% CI: 95% confidence interval.

## Data Availability

The data underlying this article will be shared on reasonable request to the corresponding author.
